# A Fine-Grained Image Classification and Detection Method Based on Convolutional Neural Network Fused with Attention Mechanism

**DOI:** 10.1155/2022/2974960

**Published:** 2022-09-14

**Authors:** Yue Zhang

**Affiliations:** Centre for Modern Educational Technology, Henan College of Police, Zhengzhou 450000, Henan, China

## Abstract

Due to the existence of attention system, people pay attention to the distinguishable area of the image, rather than directly receiving and processing the information of the whole image. This natural advantage makes attention mechanism widely used in fine-grained image classification. The research goal of fine-grained image classification task often is to differentiate subclass objects belonging to the same basic category. The difficulty of classification is that there are only slight local differences between different categories, but there may be large feature differences within the same category. At the same time, complex background features also bring interference factors to image recognition. In order to further extract discriminant regional features, this paper proposes a fine-grained image classification method WSFF-BCNN based on weak supervision feature fusion from two aspects: the improvement of the loss function in the training process of convolution neural network and the refinement of fine-grained image feature extraction. It uses the mixed attention of channel domain and spatial domain to obtain the detailed description information in the feature to highlight the response of the corresponding channel and spatial location in the feature map and pay attention to the attention characteristics of different dimensions. The original images of different sizes are input into the improved bilinear model to obtain multi-scale features. The large-scale features can represent the spatial location information of key areas, and the small-scale features represent the low-level features of the image. The backbone network of bilinear network uses ResNet50 to extract features and sample and zoom and uses bilinear pooling to fuse features of different scales to obtain a rich image feature representation.

## 1. Introduction

Accurate classification of various types of visual objects is the basis of many advanced image understanding tasks. The research of fine-grained image classification has gradually become one of the hot research directions in the field of computer vision. It mainly includes two categories: fine-grained image classification based on strong supervision information and weak supervision information [1]. The former needs to introduce additional manual annotation, and the latter can locate the key areas of the image only under the supervision of label information. Fine-grained image classification is a research direction for fine-grained image classification [2]. Compared with the coarse-grained image classification task, the classification granularity of the category to which the fine-grained image belongs is more detailed. The characteristics of different subcategories often have only slight local differences but disturbed by uncertain factors such as light, occlusion, and shooting angle; the samples of the same subcategory may have large feature differences, making fine-grained image classification a challenging task [3].

As one of the important branches of machine learning, deep learning has developed rapidly in recent years and is widely used in computer vision, speech recognition, natural language processing, and other fields. The proposal of CNN meets the needs of automatic extraction of image features, greatly improves the operation speed and accuracy of image recognition, and saves unnecessary human consumption. Applying CNN to the field of fine-grained image classification, with its powerful feature extraction ability, we can obtain more effective multi-dimensional abstract features, mine the subtle local feature information of fine-grained images, further improve the recognition efficiency and accuracy, and reduce the recognition cost. It is of great research significance to the application and development of fine-grained image recognition in various fields [4]. B-CNN (Bilinear CNN) model is an end-to-end fine-grained image classification algorithm based on feature coding. It can complete classification only by using image category tags, which belongs to the weak supervised learning method. However, the B-CNN model uses the image features extracted by two ResNet50 to complete the classification by feature fusion; the feature extractor does not capture the local regional features of the image comprehensively, and only considers the construction of a strong feature representation of the image [5, 6]. Therefore, this paper considers improving the B-CNN model from the aspect of capturing the local distinctive regional features of the image and adopts the improved ResNet50 as the backbone network of bilinear CNN, combine the proposed attention mechanism to automatically focus the space and channel, suppress the characteristics of background interference, integrate local localization into the bilinear deep learning framework, and carry out end-to-end training, so as to effectively improve the accuracy of fine-grained classification and further improve the classification performance of the model.

The main contribution of this paper is to propose a fine-grained classification scheme, which can learn useful regional features from many local features under the supervision of only label information and combine local features with semantic features to achieve effective classification. The model is composed of a target acquisition network and an image classification network. A bilinear convolution neural network based on attention mechanism is proposed. Two improved ResNet50 are used as feature extractors, which not only increase the depth of the network model but also reduce the amount of parameters. Then, an attention mechanism is proposed based on space and channel, which enables the network to automatically obtain the features of the image with distinguishing local areas and fuse the high-dimensional and low-dimensional features extracted by the two networks, respectively. With the help of the image category label, the image classification task is automatically completed, which improves the classification accuracy of the network and reduces the calculation cost.

## 2. Related Work

The fine-grained image classification method based on deep learning is divided into three ways: strong supervised learning, weak supervised learning, and unsupervised learning. The essential difference between these three ways is that the strong supervised learning method uses not only image-level labels but also bounding box and component annotation. The weak supervised learning method only uses image-level labels, while the unsupervised learning method does not use any labels for classification. When training the model, the fine-grained image classification algorithm based on strongly supervised learning needs to use the annotation box of image targets, the annotation information of target components, and image category labels [7, 8]. Extracting the component details of the target in the image is the key to the task of fine-grained image classification, so there are many algorithms using manually labeled component labels, but the labeled information needs to be made carefully and manually, and the cost is large, which hinders the application of the method relying on component label information in the actual scene under certain conditions.

Therefore, only using image category tags for classification and prediction is the research hotspot of fine-grained image classification. Attention mechanism plays an indispensable role in deep learning [9, 10]. Attention mechanism is a mode of resource allocation, which can focus on local information, invest more energy in dealing with important information, and ignore unimportant information. In recent years, the idea of attention mechanism has been widely used in various disciplines, especially in natural language processing and computer vision. Generally speaking, the attention mechanism is to get the parts that need to be mainly concerned from the image and then add a lot of attention resources to the parts that need to be mainly concerned, so as to obtain the information of the parts that need to be mainly concerned in the image [11]. At the same time, it also suppresses other irrelevant information in the image, so that we can accurately and quickly know what the main content of the painting is. From the differentiability of attention, attention can be divided into hard attention and soft attention. ResNet50 network adds attention modules to the convolution blocks of conv2 and conv3 and conv4 and conv5 of the two networks, respectively, to obtain richer and discriminative attention features [12]. Input the converged bilinear eigenvectors into the classification function to obtain the classification probability. Because the classification layer of Resnet50 network is set with 1000 categories of image classification tasks. Therefore, this paper is to remove the original classification layer and set a fixed number of neuron classification layer according to the actual needs of classification tasks [13].

Da P et al. [14] presented region-based, convolutional neural network for accurate and efficient TV logo classification. Maximally stable extremal region (MSER) was used as a method of generating candidate boxes per image. Zhang et al. [15] presented a novel method based on Random Forest classification with multi-type features to detect the logo regions on arbitrary images, and the detected logo regions were further recognized using the visual words with spatial correlated information. Druzhkov and Kustikova [16] consider such deep models as autoencoders, restricted Boltzmann machines, and convolutional neural networks. Existing software packages for deep learning problems are compared. Song et al. [17] proposed a synchronized deep autoencoder network for the simultaneous detection and classification of cells in bone marrow histology images. The proposed network used a single architecture to detect the positions of cells and classify the detected cells, in parallel. Liu and An [18] introduced the idea of sparse representation into the architecture of the deep learning network and comprehensively utilized the sparse representation of well multi-dimensional data linear decomposition ability and the deep structural advantages of multilayer nonlinear mapping to complete the complex function approximation in the deep learning model. Liu et al. [19] proposed an active learning algorithm based on a weighted incremental dictionary learning. This algorithm trained a deep network efficiently by actively selecting training samples at each iteration. Chen et al. [20] proposed the idea of deep learning ensemble framework, which was loosely based on the integration of deep learning model and random subspace-based ensemble learning. Ma et al. [21] proposed a sparse representation classification method based on the optimized kernel function to replace the classifier in the deep learning model, thereby improving the image classification effect. [22] presented a vehicle logo recognition using a deep convolutional neural network (CNN) method and whitening transformation technique to remove the redundancy of adjacent image pixels.

Backpropagation algorithm with stochastic gradient descent optimization technique had been deployed to train and obtain weight filters of the networks. Li et al. [23] aimed to minimize the user variability in training CNN by automatically searching and optimizing the CNN architecture, particularly in the field of vehicle logo recognition system. Soon et al. [24] used deep learning methods that were based on data optimization for vehicle logo in complex scenes. They proposed three augmentation strategies for vehicle logo data: cross-sliding segmentation method, small frame method, and Gaussian distribution segmentation method. Bianco et al. [25] proposed a mapreduce-based CNN called MRCNN to train the networks. Furthermore, unlike the previous classical CNN starting from a random initialization, they proposed a novel genetic algorithm global optimization and Bayesian regularization approach called GABR in order to initialize the weights of classifier. Yousaf et al. [26] developed and applied an improved deep convolutional neural network model to perform the automatic classification of breast cancer using pathological images, in which data enhancement and migration learning methods were used to effectively avoid the overfitting problems with deep learning models when they are limited by the training image sample size. Soon et al. [27] proposed two multi-view learning approaches to tackle the insufficient data issue. On one hand, a multi-view ordinal classification with a multi-view max pooling (MVMP) approach was proposed. On the other hand, in order to account for the ordinal relation, they proposed a double-task learning on MVMP for classification and average pooling for regression. Ke and Du [28] proposed a deep multi-view learning method to deal with the small sample problem of HSI. Li and Hu [29] used an end-to-end Convolutional Long Short Term Memory (ConvLSTM) network to learn the deep mutual information of polarimetric coherent matrices in the rotation domain with different polarimetric orientation angles (POAs) for unsupervised PolSAR image classification.

The feature differences of objects in fine-grained images are only reflected in local nuances. The difficulties of classification are mainly manifested in two aspects: one is to accurately locate the key areas with discrimination in the image, and the other is to extract effective features from the detected key areas and classify them. Therefore, how to effectively detect the foreground objects in the image and mine the local details of fine-grained objects is the primary problem that fine-grained image classification algorithms need to solve. Most of the early fine-grained image classification algorithms follow this process: first, find the key feature areas of the foreground object, then extract features on these areas and process them appropriately, and finally complete the training or prediction of the model through the classifier. Most of these algorithms rely on artificial feature extraction, and the description of fine-grained image features is limited, which makes the classification accuracy difficult to meet the actual needs.

## 3. Convolutional Neural Network with Attention Mechanism

This paper studies a fine-grained image classification method with high classification accuracy without additional local information annotation, extracting distinguishing features and fusing global features to achieve effective classification.

### 3.1. Channel Domain Weighted Attention Network

Taking the features extracted from the convolution layer as the input features, the input features contain multiple different feature channels, and each channel may correspond to different feature information in the image. The information contained in these channel features is different, which leads to their different importance for fine-grained image classification. The desired result is to focus only on those channels with discriminative information and weaken the influence of features without discriminative information. Channel domain attention is simply to use a weight value coefficient to express the importance of each channel feature. For the two-dimensional feature map obtained by convolution, channel attention can be expressed as a 1*∗*1 dimensional eigenvector.

The bilinear convolution network used in this paper still adopts two branch networks. However, both branch networks use the improved ResNet50 as the feature extractor, mainly to enhance the representation ability of network feature extraction. In this paper, the attention module is added between conv2, conv3 and conv4, conv5 of the two branch networks, respectively. Then, the weighted feature map processed by the attention module adopts a bilinear pooling operation, and a full connection layer is connected at the end of the network as the classification function. The number of neurons in the classification layer should be consistent with the number of categories of the classification task. Input the bilinear feature vector into the classification function to get the final result of fine-grained image classification.

For the weighted features generated by the two branches through the attention module: *F*_*sc*_ ∈ *R*^*w*×*h*×*c*^, the obtained weighted vectors are input to the next convolution block. Finally, the sum of the feature vectors obtained by network A and network B is bilinear fused

(1) $$\begin{array}{c} \varphi \left(I\right)=\sum_{I\in L}\left(F_{A}^{T}∙F_{B}\right)\text{.} \end{array}$$

Convert *φ*(*I*)=*R*^*c*×*c*^ into a one-dimensional vector and normalize the weighted bilinear vector into (2) and (3)

(2) $$\begin{array}{c} y=sign\left(\varphi \right)\sqrt{\left|\varphi \right|}\text{,} \end{array}$$

(3) $$\begin{array}{c} z=\frac{y}{{\left\|y\right\|}_{2}}\text{.} \end{array}$$

Finally, the obtained bilinear weighted vector is used as the input of the classification function to get the final classification result

(4) $$\begin{array}{c} s=\sigma \left(W_{1}z+b\right)\text{,} \end{array}$$

where *σ* represents the Softmax classification function and *b* is the offset value of the current layer.

The bilinear convolution neural network based on the attention mechanism proposed in this paper, by adding the attention module, highlights the channels that can distinguish the local regional features, suppresses the useless information interference that has little impact on the classification results, improves the accuracy of local feature location, reduces the impact of background occlusion and object deformation on the classification results, and thus improves the classification accuracy of fine-grained images.

The calculation steps of the channel domain attention are as follows:

(1) Squeezing: the feature map generated by the residual network is used as the original input *x* of the attention model, and the global average pooling method is used to compress the features of all channels into a number, expressed in *z*, then:
  (5) $$\begin{array}{c} z_{c}=\frac{1}{w\times h}\overset{h}{\underset{i=1}{\sum }}\overset{w}{\underset{j=1}{\sum }}x_{c}\left(i,j\right),x\in R^{w\times h\times c},z\in R^{c}. \end{array}$$
Among them, the dimension of *x* is *w∗h∗c*; *c* represents the channel of the feature, (*i*, *j*) represents the coordinate position on a certain channel, and after the extrusion calculation *z* is a vector of one dimension;
(2) Activation, input *z* to the full connection layer and calculate the relationship between channels;
  (6) $$\begin{array}{c} s=\sigma \left(W_{2}ReLU\left(W_{1}z\right)\right)\text{.} \end{array}$$
The bottleneck structure is used in the two fully connected layers. An FC pair is used to reduce the dimension *z*. The dimension reduction ratio is 1/*r*; *r*is the dimension reduction coefficient, and then activated it using Re L U, and an FC is used to increase the dimension. The parameters of the two fully connected layers are expressed by *W*_1_ and *W*_2_, respectively. *s* belongs to *R*^*c*^. The dimension of *s* is equal to *z*. *σ*(∙) indicates sigmoid activation;
(3) Sigmoid activation function normalizes the channel weight value to (0, 1) and multiplies the activated weight value with the original feature points to obtain the weighted feature map as $\bar{x}\in R^{w\times h\times c}$.

### 3.2. Spatial Domain Weighted Attention Network

**Figure 1:**
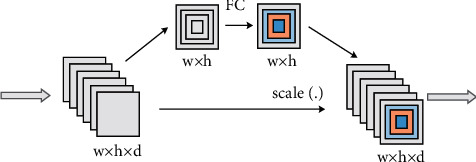
Channel domain attention network module.

Spatial attention pays more attention to the information on the spatial position of the discriminative region of the feature map. It can be said that spatial attention is a supplement to channel attention. The schematic diagram of the spatial attention module is shown in Figure 1 [30].

The pixel values of each spatial position in the feature map are taken out separately and combined into a one-dimensional feature vector. The spatial domain attention mechanism refers to learning a weighted real number for all one-dimensional features in these spatial positions to represent the importance of these features. For the entire three-dimensional feature map, the weight that spatial domain attention needs to learn is a two-dimensional weight matrix corresponding to the size. Through the learning of spatial attention, the feature response at the significant local spatial position in the feature map will continue to increase. The spatial attention module is added to the residual network. The specific process of feature extraction and fusion of attention module is as follows:

1. The feature function extracts the feature map *F*, *F* ∈ *R*^*w*×*h*×*c*^, where *w∗h* is the spatial dimension of the feature map, *c* indicating the number of channels.
2. Global average pooling *P*_*a*_ and global maximum pooling *P*_*m*_ are used to reduce the dimension of the feature map *F*. The obtained two feature maps *w∗h∗*1 are spliced by summing the corresponding elements, and useful information is extracted through a full connection layer.
3. Using a 7 × 7 convolution kernel convolutes the characteristic image obtained in the previous step, and the size is compressed to *w∗h∗*1. Then, use the sigmoid activation function to map and generate the spatial attention map *A*_*s*_, *A*_*s*_ ∈ *R*^*w*×*h*×1^.
4. The method of point multiplication by element is used to fuse the features of *F* and *A*_*s*_to generate the final spatial attention feature map *F*_*s*_, *F*_*s*_ ∈ *R*^*w*×*h*×*c*^.
5. Through global average pooling *P*_*a*_ and full connection layer learning of spatial attention feature map*F*_*s*_, the descriptors of all output feature maps are obtained, with the size of 1 × *c*.
6. Use the activation function ReLU to allocate the channel attention weight and generate the channel attention weight *A*_*c*_, *A*_*c*_ ∈ *R*^1×*c*^.
7. The feature fusion of *F*_*s*_ and *A*_*c*_ is carried out by the method of corresponding element point formation, and the key extraction of channel dimension is realized. Finally, a multi-dimensional attention feature map *F*_*sc*_ is generated, *F*_*sc*_ ∈ *R*^*w*×*h*×*c*^.

Combining the two dimensions of attention, it has a stronger local feature extraction ability, so that the network can obtain more abundant features. Through multi-dimensional attention, we can avoid the loss of important information when extracting features from the network. Add the attention module, first obtain the local areas with discriminative information with spatial attention, and then distinguish the channels corresponding to the local features of different subcategories through the channel attention retention significant high response. The two promote each other. After weighting the feature map, it is input to the Softmax classification layer to get the category of the image and realize the classification task.

### 3.3. Fine-Grained Classification Based on Attention Mechanism and Multi-Scale Features

The difference between different subclasses of fine-grained images is small and difficult to detect; different individual images of the same subcategory have large intra-class differences due to the influence of posture, angle, background, and so on. In order to strengthen the attention to distinguishing local regional features, this paper designs a classification method that integrates attention mechanism and multi-scale features and improves it. ResNet50, a residual network with more layers of features and stronger expression ability, is used to extract global features and local features, respectively, and the upper and lower networks interact. By adding the attention mechanism of channel domain and spatial domain to the residual structure, filtering out the irrelevant channels in the feature map, and focusing on the local region of spatial response, we get rich multi-dimensional attention features. Automatically focus on key parts without using additional manual labels to improve the classification effect of the network.

#### 3.3.1. Multiscale Feature Fusion

**Figure 2:**
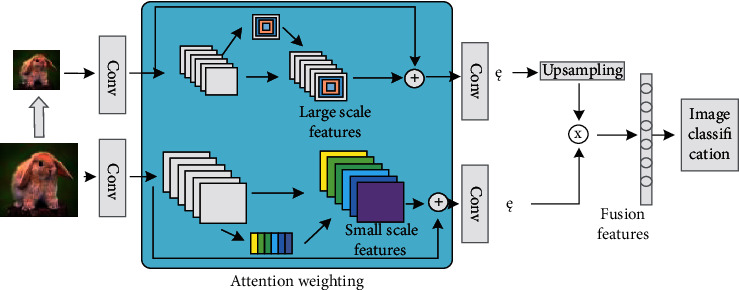
Overall network framework.

Through the multi-level feature combination to enrich the feature representation of the significant region, obtain the key part features while retaining the global features and get rich multi-level features. In the designed network model, it is also to improve the representation ability of features and fuse multi-scale features. The two inputs of the bilinear network correspond to the original graph. Multiscale feature fusion network model is shown in Figure 2.

The network convolutes the input images of different sizes with the scaled sample image of the original image to obtain the characteristic images of different sizes. Each position of the feature map output by the network corresponds to different sizes of regions in the original image, and the feature map of different scales can represent the features of different sizes in the image.

When the convolution kernel is in the same size, the large-scale features often correspond to the larger region in the original image, and the small-scale features correspond to the smaller region. They describe the global information and detailed information of the image, respectively. In the task of fine-grained image classification, large-scale features are used to represent the key parts, and small-scale features are used to represent the low-level information of these parts. By fusing the features of different scales, more expressive features can be combined, which is more conducive to fine-grained image classification.

Multi-scale features can be obtained by controlling the size of the input image. Before fusing different size features, the two feature images need to be unified into the same size. The bilinear interpolation method is used to sample the small-size feature map, enlarge it to the size of the large-size feature map, and make full use of the information in the two feature maps.

#### 3.3.2. Attention Characteristics

This paper analyzes the fusion of channel attention and spatial attention features and uses ResNet50 as a feature extractor to add channel domain attention to the residual block of the upper network and spatial domain attention to the residual block of the lower network. Through the two kinds of attention, the salient parts of images with different sizes are detected, and the features are extracted, respectively, and finally combined them. ResNet50 is composed of 50 convolution layers, which contain five stages, which are represented by stagel1-stage5, respectively. Each stage2-stage5 also contains three, four, six, and three residual blocks. If the size of the image input by ResNet50 is 224*∗*224*∗*3, the feature size of the convolution output of the last layer is 7*∗*7*∗*2048, the size of the feature image is reduced by half on each stage, and the number of channels is doubled.

After using the residual network to obtain the weighted feature maps in the channel domain and spatial domain, we can use bilinear interpolation to unify the size of these two different sized attention maps and then use bilinear pooling to fuse features. The channel attention characteristic map can be expressed as *M*_*c*_, and the spatial attention characteristic map can be expressed as *M*_*s*_, then:

(7) $$\begin{array}{c} M_{f}={\left(\varphi \left(M_{c}\right)\right)}^{T}M_{s}\text{,} \end{array}$$

*M*_*f*_ represents the fusion result of the two feature maps, and *φ*(∙) represents the bilinear interpolation operation. Adding multiple channel domain and spatial domain attention modules to the residual network can focus on the salient features from different dimensions.

## 4. Improved Loss Function

### 4.1. Improvement of Classification Function

The loss function of target detection task consists of classification loss and regression loss. The loss function is divided into the following four parts: confidence loss Loss_conf_, category loss Loss_class_, coordinate loss Loss_*xy*_ and width height loss Loss_*wh*_. The formula is as follows:

(8) $$\begin{array}{c} Loss\left(object\right)={Loss}_{xy}+{Loss}_{wh}+{Loss}_{conf}+{Loss}_{class}\\ =\lambda \overset{K*K}{\underset{i=0}{\sum }}\overset{M}{\underset{j=0}{\sum }}I_{ij}^{obj}\left(2-w_{i}\times h_{j}\right)\left[{\left(x_{i}-{\hat{x}}_{i}\right)}^{2}+{\left(y_{i}-{\hat{y}}_{i}\right)}^{2}\right]\\ \begin{array}{c} +\lambda \overset{K*K}{\underset{i=0}{\sum }}\overset{M}{\underset{j=0}{\sum }}I_{ij}^{obj}\left(2-w_{i}\times h_{j}\right)\left[{\left(w_{i}-{\hat{w}}_{i}\right)}^{2}+{\left(h_{i}-{\hat{h}}_{i}\right)}^{2}\right]\\ \begin{array}{c} -\overset{K*K}{\underset{i=0}{\sum }}\overset{M}{\underset{j=0}{\sum }}I_{ij}^{obj}\left[{\hat{C}}_{i}\log\left(C_{i}\right)+\left(1-{\hat{C}}_{i}\right)\log\;\left(1-{\hat{C}}_{i}\right)\right]\\ -\beta \overset{K*K}{\underset{i=0}{\sum }}\overset{M}{\underset{j=0}{\sum }}I_{ij}^{noobj}\left[{\hat{C}}_{i}\log\left(C_{i}\right)+\left(1-{\hat{C}}_{i}\right)\log\;\left(1-{\hat{C}}_{i}\right)\right]\\ -\overset{K*K}{\underset{i=0}{\sum }}I_{ij}^{obj}\underset{c\in class}{\sum }\left[{\hat{p}}_{i}\log\;\left(p_{i}\left(c\right)\right)+\left(1-{\hat{p}}_{i}\left(c\right)\right)\log\;\left(1-{\hat{p}}_{i}\left(c\right)\right)\right]. \end{array} \end{array} \end{array}$$

The loss function Loss_*xy*_ is expressed as (*x*_*i*_, *y*_*i*_) coordinate error, the second item Loss_*wh*_ is the width height coordinate error, WI, *h*_*i*_ is the length and width of the *ith* grid prediction frame, *k∗k* is the number of grids, *M* is the candidate boxes generated by the grid, and each candidate box will get the corresponding bounding box through the network, and finally get *k∗k∗m* bounding boxes. The confidence error Loss_conf_ is calculated using cross-entropy, using cross-entropy loss. However, the original cross-entropy loss function will reduce the recognition accuracy in the case of unbalanced samples, and there are more small targets and fewer targets in the target statistics of sample data. Therefore, the prominent challenge of target detection is the extreme imbalance between positive and negative samples and difficult samples. In order to solve the problem of sample imbalance, focal loss is introduced to replace the original cross-entropy classification loss function. The formula is as follows:

(9) $$\begin{array}{c} Focal\;loss=\left\{\begin{array}{cc} -\alpha {\left(1-y^{\prime }\right)}^{\beta }\;\log\;y^{\prime }\text{,} & y=1\text{,}\\ -\left(1-\alpha \right){y^{\prime }}^{\beta }∙\log\;\left(1-y^{\prime }\right)\text{,} & y=0\text{,} \end{array}\right. \end{array}$$

where *y* ∈ {±1}is the real category, and the probability value between *y*′ ∈ {0,1} is the model probability estimated by the activation function. In addition, focal loss introduces two hyperparametric sums *α* and *β*, in which the balance factor *α* represents the corresponding weights of different categories, which is equivalent to weighting the positive and negative samples, so as to balance the positive and negative samples; The attenuation coefficient *β* > 0, as the loss of samples, pays more attention to difficult samples. The larger the attenuation coefficient *β* is, the greater the attenuation degree of samples with the correct focus loss classification, which also makes its loss pay more attention to difficult and misclassified samples.

### 4.2. Improvement of Regression Function

The target detection task is to detect the position and category of the target in the image. The most commonly used evaluation index is average accuracy (MAP), and the index of accuracy between the prediction box and the real box is intersection union ratio (IOU). L2 regression loss function is the original regression loss function L1 norm loss function, also known as the minimum absolute deviation (LAD), which minimizes the sum of the absolute differences between the target value *y* and the estimated value. The L2 norm loss function, also known as least-squares error (LSE), minimizes the sum of squares of the differences between the target value and the estimated value. Formula:

(10) $$\begin{array}{c} L_{1}\left(\hat{y},y\right)=\overset{m}{\underset{i=0}{\sum }}\left|y^{\left(i\right)}-{\hat{y}}^{\left(i\right)}\right|\text{,}\\ L_{2}\left(\hat{y},y\right)=\overset{m}{\underset{i=0}{\sum }}{\left(y^{\left(i\right)}-{\hat{y}}^{\left(i\right)}\right)}^{2}\text{.} \end{array}$$

The original regression function uses the L2 loss function for bounding box regression, but this kind of loss function does not always reflect the positioning accuracy well. Therefore, in the process of target detection, IOU will be considered to measure the target positioning loss directly, and the IOU loss formula is expressed as

(11) $$\begin{array}{c} IoU=\frac{B^{p\;d}\cap B^{gt}}{B^{p\;d}\cup B^{gt}}\text{,} \end{array}$$

where *R*_CIOU_ is the penalty term of the prediction box *B*_*p* *d*_ and target box *B*_*gt*_. The original IOU-based loss function is defined as

(12) $$\begin{array}{c} L_{CIoU}=1∙IoU\text{.} \end{array}$$

Although IOU loss solves the two major problems that smooth L1/L2 series variables are independent of each other and do not have scale invariance, IOU loss only works when the bounding boxes overlap and will not provide any moving gradient in the case of non-overlap. CIOU loss, based on IOU, introduces the minimum circumscribed rectangle of the prediction box and real box, which can solve the gradient optimization problem of a disjoint rectangular box, but there are still some limitations in training and convergence speed. In this paper, CIOU loss is added to replace the original regression function to achieve more accurate prediction box regression. The overall formula is expressed as

(13) $$\begin{array}{c} L_{CIoU}=1∙IoU+R_{CIoU}\left(B_{p\;d},B_{gt}\right)\text{.} \end{array}$$

The difference between CIOU_Loss_ and IOU loss is that three geometric factors, including overlapping area, center point distance, and aspect ratio, are considered in the boundary box regression to solve the inconsistency between the measurement function and the boundary regression mechanism in the process of target detection. In CIOU_Loss_, the specific settings are:

(14) $$\begin{array}{c} R_{CIoU}=\frac{\varphi^{2}\left(b,b^{gt}\right)}{c^{2}}+\alpha v\text{,} \end{array}$$

where *b* and *b*^*gt*^, respectively, represent the distance between the center points of the prediction frame and the target frame, *φ*(*·*) represents the Euclidean distance between two points, and *c* represents the shortest distance of the diagonal between the two frames, while taking into account the overlapping area and the center point distance.

In addition, the parameters *α* and *v* take the aspect ratio between the two boxes into account, and the formula is as follows:

(15) $$\begin{array}{c} v=\frac{4}{\pi^{2}}{\left(\arctan\frac{w^{gt}}{h^{gt}}-\arctan\frac{w}{h}\right)}^{2}\text{,}\\ \alpha =\frac{v}{\left(1-IoU\right)+v}\text{.} \end{array}$$

The final regression function used for classification detection is

(16) $$\begin{array}{c} L_{CIoU}=1-IoU+\frac{\varphi^{2}\left(b,b^{gt}\right)}{c^{2}}+\frac{v}{\left(1-IoU\right)+v}∙\frac{4}{\pi^{2}}{\left(\arctan\frac{w^{gt}}{h^{gt}}-\arctan\frac{w}{h}\right)}^{2}\text{.} \end{array}$$

## 5. Experimental Results and Performance Evaluation

**Figure 3:**
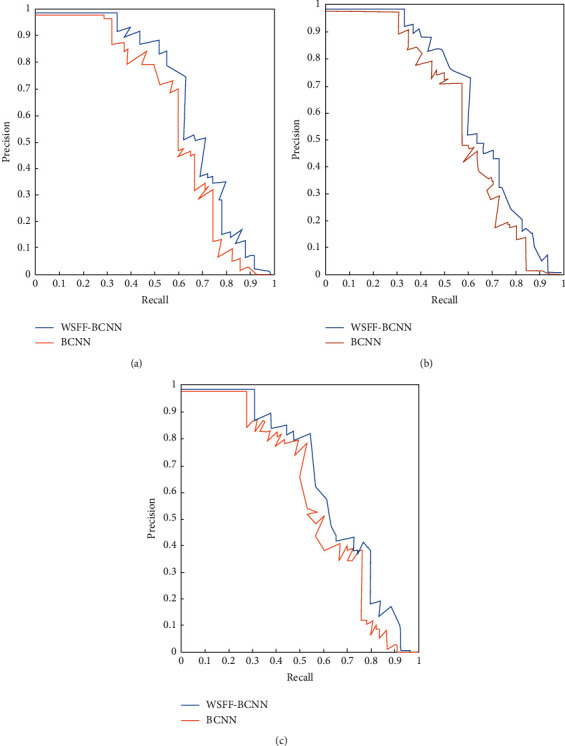
Comparison of PR curve (CUB-200-2011). (a) CUB-200-2011 2000, (b) CUB-200-2011 4000, and (c) CUB-200-2011 6000.

The data sets used in the experiment are fine-grained public data sets: CUB-200-2011 data set. The CUB-200-2011 data set has 200 different bird categories, with a total of 11788 images. Each category covers different angles, different scenes, and different postures, including 5794 test pictures and 5994 training pictures. A comparison of the PR curve is shown in Figure 3.

The experimental process is divided into two stages: in the first stage, the data are input into two networks and the two networks work in parallel at the same time. All the convolution layer parameters in front of the attention module are fixed. The parameters of the attention module are initialized randomly, and then only the parameters of the attention module and the full connection layer are trained. Finally, the classification is completed and the parameters when the classification performance of the model is the best are saved. In the second stage, the whole model is initialized with the optimal parameters saved in the previous stage, and then the parameters of all layers in the network are fine-tuned to obtain the classification accuracy and model parameters of the final model. Since the full connection layer is used, it is necessary to set the size of the input data. In the experiment, the data input into the network is 448 × 448. The SGD optimizer with a momentum of 0.9 and a weight attenuation coefficient of 0.00001 was used in the experiment. Setting of learning rate: in the first stage, the initial learning rate of the full connection layer is 0.9, and the initial learning rate of the attention module is 0.09, and the initial learning rate of the second stage is 0.001.

It can be seen from Figure 3 that the number of categories gradually increases from 2000 to 4000 and 6000. Compared with BCNN, the WSFF-BCNN model achieves better performance than other benchmarks on data sets of different sizes. This paper further calculates the “precision” and “recall” of BCNN and WSFF-BCNN to illustrate the accuracy and missed detection rate of detection. The larger the area surrounded under the curve, the better the detection performance. It can be seen that WSFF-BCNN significantly improves the recall rate, which shows that this method alleviates the lack of small objects in target detection.

**Figure 4:**
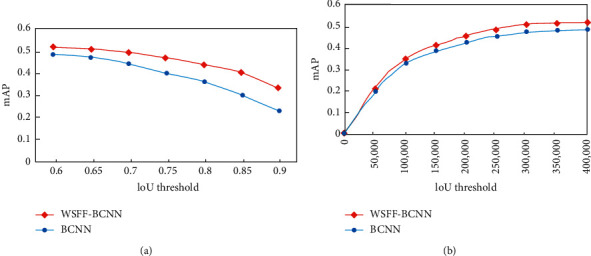
Sensitivity of parameters. (a) IoU threshold and (b) IoU threshold.

This paper evaluates the detection performance by changing different IOU thresholds from 0.6 to 0.9 at 0.05 intervals. As shown in Figure 4(a), when changing the IOU threshold, WSFF-BCNN (red curve) has a more stable performance improvement than BCNN (blue curve). This paper also sets different iteration times to compare the convergence and accuracy of the model.  Figure 4(b) shows that with the increase of the number of iterations, the performance is gradually improved, and the accuracy is higher than that of BCNN.

Obviously, compared with the BCNN method, WSFF-BCNN can detect some targets that are not easy to detect, such as occluded, blurred, and small targets. The method proposed in this paper has great advantages in small area detection, which shows the effectiveness of multi-scale feature fusion.

**Figure 5:**
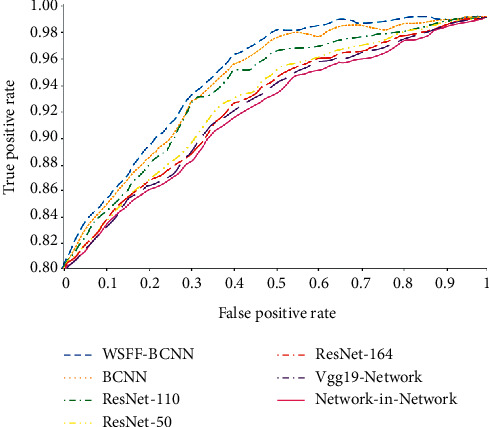
ROC classification curve.

The ROC curves of all methods are drawn in Figure 5 to show the stability of each method. The larger the area below the curve, the more stable its classification performance is, which shows that the performance of this method is the best. Compared with other methods, the classification difficulty increases with the increase of categories, and the prediction accuracy is still very high.

The improved model in this paper solves some limitations of the original B-CNN model. For the input image, the specific location of the target object is obtained through network to reduce the interference of background information. Compared with the network of the original model, the improved ResNet50 is used as the feature extractor to enhance the ability of feature representation, improve the training speed of the network, and reduce the phenomenon of overfitting. Adding the attention module to the residual network has a stronger ability of the local feature extraction and richer feature representation. The high-dimensional and low-dimensional features extracted by the two networks are fused with bilinear features, so that the proposed bilinear convolution neural network based on attention mechanism pays more attention to the more discriminative local region features in learning and training and improves the classification accuracy of the whole network.

## 6. Conclusion and Future Work

There are many difficulties in fine-grained classification task, which leads to poor classification effect. There are only subtle feature differences among various types of fine-grained images. How to find and use these local area information is the problem that fine-grained image algorithm needs to solve. Based on the relevant research of CNN, this paper studies the fine-grained image feature extraction and thinning and the improvement of loss function in the training process of convolutional neural network. Firstly, the features extracted by the traditional CNN algorithm in the feature extraction stage cannot capture the location of the local region of the image, which makes the model unable to pay full attention to the local differentiated region of the target. This paper designs a WSFF-BCNN model based on the convolution attention module, which proves that the attention mechanism can capture the local area position of the image target, so as to improve the recognition ability of the model. Through relevant experiments on the open data set in the fine-grained field, it shows better advantages. Compared with other models, it proves the effectiveness of the WSFF-BCNN model proposed in this paper, the effect is better, and it is more suitable for the target detection task in the real scene.

In addition, although this paper solves the problems of large-scale classification, multi-scale detection, and effective feature extraction in images to a certain extent, due to the diversified development of various images, the algorithm should be further optimized. Although the training method proposed in this paper is an effective classification with label information, it is still a supervised method in essence. Therefore, the next step can focus on the unsupervised fine-grained classification. At the same time, in the detection task, we still face severe challenges such as multi-label and large-scale problems. Although the method proposed in this paper effectively improves the effect of target detection, it still needs further research on target classification and detection in the future.

## Data Availability

The authors confirm that the data supporting the findings of this study are available within the article.
